# Clinically Uncertain Liver Masses: A Guide to Distinguishing Poorly Differentiated Primary Liver Cancer

**DOI:** 10.3390/biomedicines13051063

**Published:** 2025-04-28

**Authors:** Greta Sökeland, Michael P. Brönnimann, Erik Vassella, Guido Stirnimann, Matteo Montani, Juliane Friemel

**Affiliations:** 1 Institute of Tissue Medicine and Pathology, University of Bern, 3008 Bern, Switzerland; 2 Department of Neurology, Bern University Hospital, 3010 Bern, Switzerland; 3 Department of Diagnostic, Interventional and Pediatric Radiology, Bern University Hospital, 3010 Bern, Switzerland; 4 Department of Hepatology, Inselspital, Bern University Hospital, 3010 Bern, Switzerland; 5 Institute of Forensic Medicine, University of Zürich, 8057 Zürich, Switzerland

**Keywords:** poorly differentiated primary liver cancer, uncertain liver mass, *TERT* mutation, HepPar1, Arginase-1, consensus tumor board

## Abstract

**Objectives**: The distinction of clinically uncertain, poorly differentiated liver masses into primary liver cancer (PLC) of cholangiocytic origin (intrahepatic cholangiocarcinoma; CCA) or hepatocellular origin (hepatocellular carcinoma; HCC) vs. metastasis is highly relevant to guiding patient treatment. Protocols differ in terms of resection, local ablation, liver transplantation, or systemic therapies with immune checkpoint inhibitors (ICIs) and tyrosine kinase inhibitors (TKIs). **Methods**: This retrospective case series exemplifies a multidisciplinary, practical guide to clinically uncertain liver masses using imaging, histomorphology, immune phenotyping, and mutational testing of telomerase promoter (*TERT*) combined with a literature review. **Results**: In 2/3 patients with uncertain liver masses and inconclusive immunohistochemistry profiles, *TERT* testing supported the diagnosis of poorly differentiated hepatocellular carcinoma. The third case with a history of sclerosing cholangitis and vague adenoid morphology yielded mutations in *ARID1a* and *TP53* and was identified as primary liver cancer, consistent with poorly differentiated intrahepatic cholangiocarcinoma or mixed hepatocellular cholangiocarcinoma (cHCC/CCA). **Conclusions**: Finding HCC-typical *TERT* promoter mutations is a useful diagnostic tool in poorly differentiated primary liver cancer.

## 1. Introduction

Sorting clinically uncertain liver masses into hepatocellular carcinoma (HCC), intrahepatic cholangiocarcinoma (iCCA), or metastasis in the setting of CUP (cancer of unknown primary) can be difficult when neoplastic cells are poorly differentiated and immunohistochemistry markers show inconclusive phenotypes. Broad-panel sequencing is often not feasible upfront. Metastases are by far the most frequent reason for liver nodules. Primary liver cancer (PLC) comprises hepatocellular carcinoma (HCC; 75–85% of cases), intrahepatic cholangiocarcinoma (iCCA; 10–15% of cases), and combined hepatocellular-cholangiocarcinoma (cHCC/CCA; <5%) [1]. The distinction is clinically relevant, because treatment protocols for HCC patients include surgical resection, local ablation, liver transplantation, or systemic therapies with immune checkpoint inhibitors (ICIs) and tyrosine kinase inhibitors (TKIs) [2]. In contrast, patients with intrahepatic CCA are rarely eligible for surgical resection (~25% of cases) and survival benefit after liver transplantation has been reported as unsatisfactory [3]. Patients with unresectable tumors are submitted to undergo palliative systemic chemotherapy with gemcitabine and cisplatin [4]. This retrospective case series exemplifies a practical guide to clinically uncertain liver masses using imaging, histomorphology, and immune phenotyping supplemented with the mutational testing of telomerase reverse transcriptase promoter (*TERT*).

**Figure 1:**
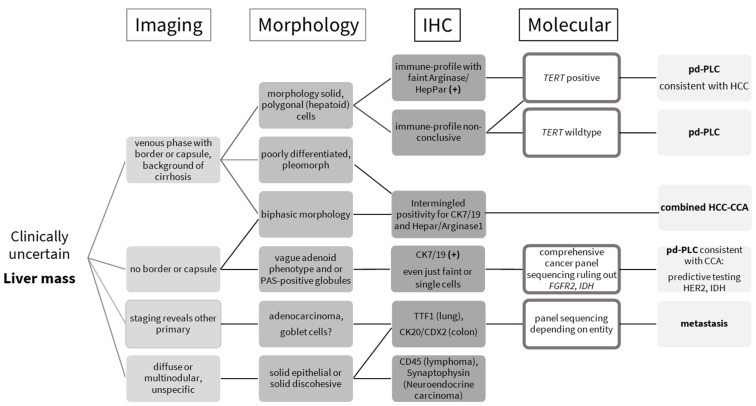
Practical guide to clinically uncertain liver masses, essentially combining morphology, immunohistochemistry, and molecular testing. *TERT*—telomerase reverse transcriptase promoter; pd-PLC—poorly differentiated primary liver cancer; HCC—hepatocellular carcinoma; combined HCC-CCA—combined hepatocellular cholangiocarcinoma; CCA—cholangiocarcinoma.

In our center, patients with suspect liver masses receive contrast-enhanced computed tomography (CT) or magnetic resonance imaging (MRI), as recommended in international guidelines [5] (Figure 1). If the patient is eligible for biopsy, tissue analysis consists of morphology, immunohistochemistry, and molecular testing if applicable. Broad-panel sequencing is only performed if there is a pre-test probability of detecting cholangiocarcinoma with predictive *FGFR* or *IDH* alterations. The main reason is the lack of therapeutic targets in hepatocellular carcinoma. All findings are discussed in the interdisciplinary liver tumor board to determine the treatment regimen.

Tissue marker expression analysis (Table 1) is performed to rule out the liver metastasis of other primary tumors (gastrointestinal tract, lung, breast, female and male genital tract). This always occurs in cooperation with radiology/staging. Poorly differentiated lesions might not exhibit specific marker profiles. Once a primary liver lesion is determined to be the most likely, cholangiocytic vs. hepatocellular origin can be further evaluated for vague morphological hepatoid or adenoid features, as suggested by Kikuchi et al. [6]. Single-target testing of telomerase reverse transcriptase promoter (*TERT*) is helpful for diagnosis if cost- and time-intensive genomic sequencing is restricted for technical or economic reasons. Approx. 70% of HCC bear *TERT* mutations. The rate of occurrence ranges from 31 to 80% [7,8,9,10]. *TERT* constitutes the catalytic unit of the telomerase that maintains the length of telomeres [11]. Most normal tissues have no telomerase activity, except germ cells of the ovary or testis, and display weak telomerase activity in proliferative cells of renewal tissues [12]. Malignant cells reactivate the telomerase to escape replicative telomere reduction during aging, thereby facilitating unlimited proliferation [13]. This reactivation is often brought about by *TERT* promoter mutations, which induce telomerase transcription [14]. In HCC specimens, mutations generally occur very early during tumor evolution at two main hotspots [15]. The more frequent one occurs at −124 bp from the *TERT* transcription start site, and is characterized by either a G to A, or G to T, substitution. The second hotspot at −146 bp is defined by G to A substitution [8]. In contrast, *TERT* mutations are rare in intrahepatic CCA; larger studies have reported mutations in 0–8% of cases [16]. *TERT* mutations might occur in gliomas and papillary thyroid carcinoma, two entities that rarely metastasize into the liver [17,18].

## 2. Materials and Methods

Three patients with clinically uncertain liver masses are reported. Imaging studies were performed as contrast-enhanced CT or MRI scans, but radiology left diagnosis open. Other primaries could not be detected. Biopsies were performed as recommended for non-cirrhotic patients or indeterminate nodules > 1 cm in size [5,33]. The tissue samples were formalin-fixed, paraffin-embedded, and cut into 3 µm slides (liver tumor protocol: hematoxylin–eosin, elastica–van-Gieson, periodic–acid shift). Case 1 was a liver segment resection specimen; Case 2 and 3 were transjugular liver biopsies (both < 1 cm in length) with limited amounts of tissue. The morphology revealed solid growth and poor differentiation (G3) according to the 5th edition of the WHO classification [34]. Based on the work by Kikuchi et al., we further categorized samples as polygonal/hepatoid, pleomorph, or vaguely adenoid [6]. Immunohistochemistry staining included a minimum panel of hepatocyte-specific Arginase1 (sensitivity > 90%, specificity 94%), HepPar1 (sensitivity > 90%, specificity 97%), and cholangiocyte CK7/CK19 (Table 1) [35]. For Arginase1, we used clone SP156 (dilution 1:100), a monoclonal rabbit antibody (CellMarque, Rocklin, CA, USA). For HepPar1, we used OCH1E5 at a dilution of 1:200 (Roche Diagnostics, Rotkreuz, Switzerland). CK7 had the following parameters: clone OV-TL12/30 and dilution 1:800. Searching for BAP1 (BSB-109, BioSB, Santa Barbara, CA, USA), Glypican (1G12, CellMarque, Rocklin, CA, USA), and AFP (polyclonal, Dako, Santa Clara, CA, USA) expression supplemented the panel with HCC- and CCA-specific alterations. Only the resection specimen could be further stained for other primary origins: TTF1 (SPT24, Novacastra, Biosystems, Muttenz, Switzerland), PAX8 (RM436, BioSB, Santa Barbara, CA, USA), PSA (polyclonal, Dako, Santa Clara, CA, USA), CK20 (Ks20.8, CellMarque, Rocklin, CA, USA), CDX2 (EPR2764Y, CellMarque), HMB45 (monoclonal, CellMarque), MelanA (A103, Dako), SOX11 (MRQ-58, CellMarque), CD45 (PD7/26, 2B11, Dako), CD10 (56C6, Novacastra). This was because of continuous uncertainty about other primaries.

*TERT* promoter sequencing was conducted as simple targeted Sanger sequencing in 2 cases (Primer: Forward 5-CACCCGTCCTGCCCCTTCACCTT-3; Reverse 5-GGCTTCCCACGTGCGCAGCAGGA-3). Mutational analysis of the TERT promoter hot-spot regions, located 124 and 146 bp upstream of the ATG start site, was conducted by PCR followed by Sanger sequencing. The specimen of case 3 was directly submitted to broad-panel sequencing (tumor board consensus) because of the clinical presentation with a history of sclerosing cholangitis and imaging features. Sequencing analysis was performed using the TruSight Oncology 500 (TSO500) panel; https://www.illumina.com/, NovaSeq 6000 platform (Illumina, San Diego, CA 92122, USA), which targets exonic and splice site regions of 523 cancer-related genes. Paired-end sequencing was carried out at the Clinical Genomics Lab of the Inselspital, Bern. The threshold for VAF detection was set at 5%, with an exon coverage of >500×.

The local ethics committee (Kantonale Ethikkommission Bern) approved the report of this retrospective case series. All patients signed a general consent form. Informed consent was waived due to the observational nature of the study (Req-2021–01336).

## 3. Results

**Figure 2:**
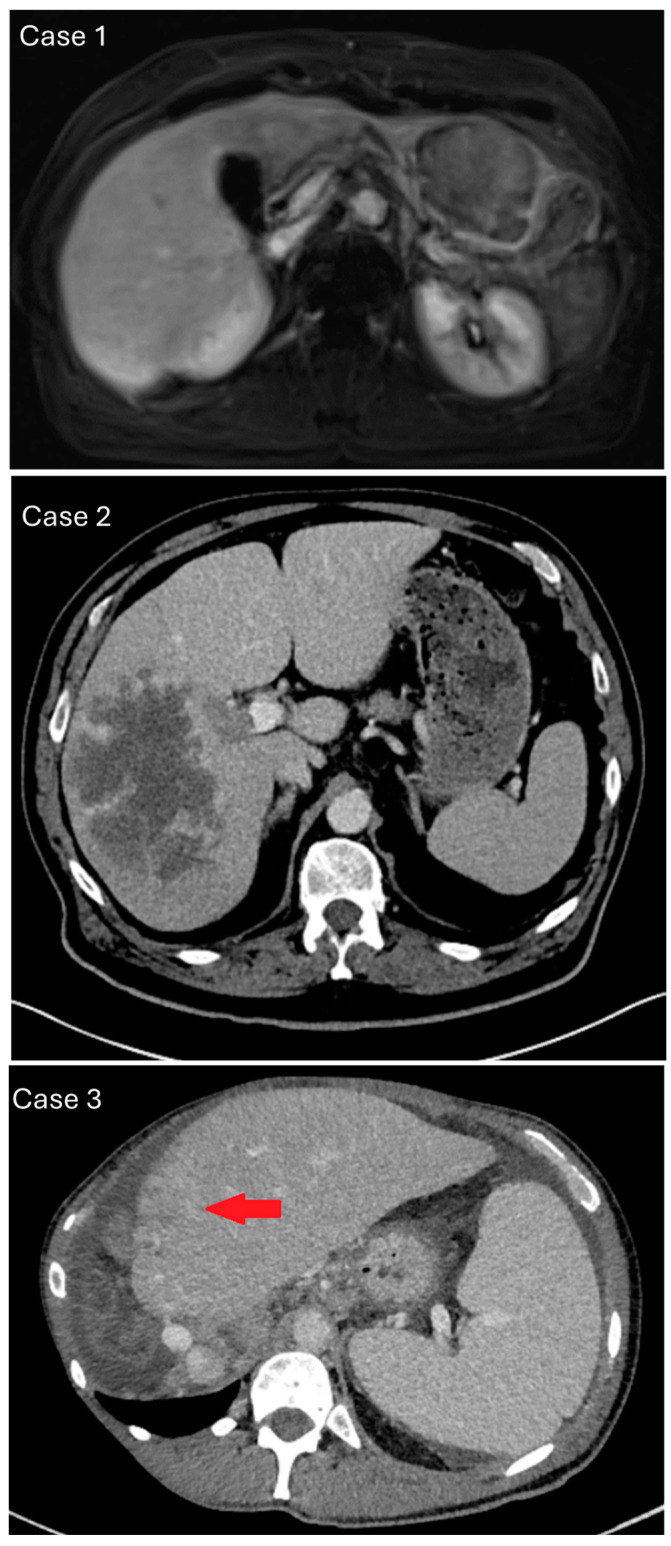
Representative chronological imaging of the case series. Upper panel/case 1: contrast-enhanced magnetic resonance imaging, arterial rim enhancement of approx. 6 cm large, suspicious liver lesion in segment II/III without CCA-typical capsular retraction. Middle panel/case 2: contrast-enhanced computed tomography, venous phase with cauliflower-like mass of right liver without border or capsule, suggesting intrahepatic cholangiocarcinoma (iCCA). Recognizable macrovascular infiltration, which is more common in hepatocellular carcinoma. Lower panel/case 3: contrast-enhanced computed tomography and a new, rim-enhancing mass in liver segment II with capsular retraction and signs of macrovascular infiltration (red arrow).

Case 1 was a 71-year-old female with a suspect lesion in liver segment II/III without evidence of chronic liver disease. There was neither detection of tumor marker alpha-fetoprotein (AFP), CA19–9, nor CEA. Contrast-enhanced magnetic resonance imaging showed a 6 cm large, suspicious liver lesion in segment II/III without capsular retraction and a central enhancement in the late venous phase. This was more distinct for cholangiocarcinoma (Figure 2; upper panel). The tumor board consensus was to perform laparoscopic surgical resection.

**Figure 3:**
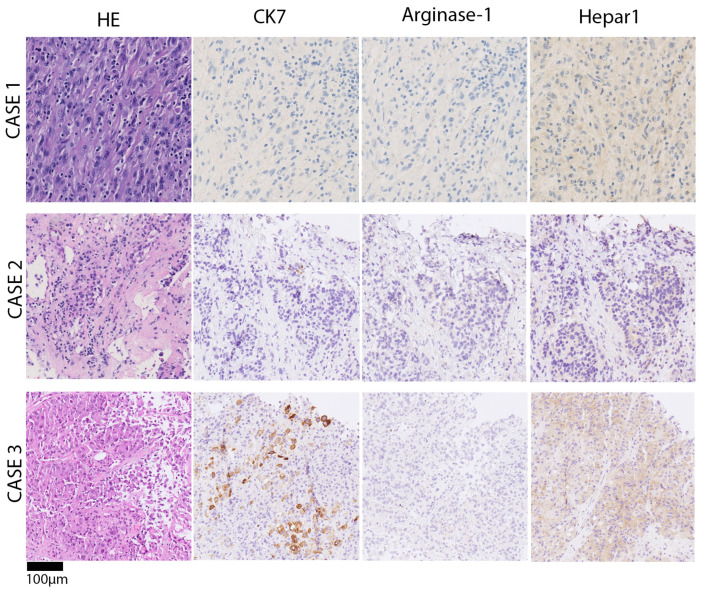
Chronological presentation of histomorphology and representative immune phenotypes of case series. Upper panel/case 1: poorly differentiated carcinoma, solid growth pattern, lymphocyte-infiltrate, and necrosis. Very faint positivity for hepatocyte paraffin-1 (HepPar1), which was initially interpreted as unspecific. Middle panel/case 2: poorly differentiated carcinoma, solid growth pattern, and necrosis in the right liver. Immunohistochemistry showed focal very faint HepPar1 positivity in tumor cells, Arginase-1 and CK7 negativity. Lower panel/case 3: poorly differentiated carcinoma with a predominantly solid, partial trabecular growth pattern and necrosis. Vague adenoid features, i.e., intracytoplasmatic globules. Single-cell positivity for CK7 and faint HepPar1 positivity.

In a liver segment II/III resection specimen (278 g, 18 × 9 × 5 cm), we observed a whitish-yellowish tumor, max. 7 cm in diameter, with multiple adjacent satellite nodules, max. 1.9 cm in diameter. Histology revealed a poorly differentiated carcinoma with a predominantly solid growth pattern with a mesenchymal appearance, lymphocyte infiltration, and necrosis (Figure 3). Tumor cells showed focal intracellular iron storage. The surrounding non-tumorous liver parenchyma had no significant fibrosis and minimal steatosis. Specimen staining showed faint positivity for hepatocyte paraffin-1 (HepPar-1). Arginase 1 was negative and CK7 < 5%. Unspecific markers like CD10, CK18, and PanCK were positive. Negative staining involved GS-6, EBER, CD117, DOG1, ERG, CDX2, SF1, Inhibin, Glypican-3, MelanA, HMB45, SOX10, and TTF1. BAP1 nuclear staining was preserved, indicative of BAP1 retention. *TERT* mutation analysis through Sanger sequencing yielded a *TERT* mutation at the hotspot 228 C > T (−124 bp). In summary, the combination of *TERT* mutation and faint HepPar-1 staining made us identify the case as poorly differentiated primary liver cancer, consistent with poorly differentiated hepatocellular carcinoma. The patient received adjuvant chemotherapy with Capecitabin and later a cisplatin/gemcitabine regimen because of tumor recurrence. The overall survival was 43 months.

Case 2 was a 72-year-old male with a suspect lesion in the right liver and a smaller one in the left lateral (Figure 2; middle panel). There was a moderate rise in tumor marker alpha-fetoprotein, which went from 223 to 310 ng/mL within one month. CT imaging showed a cauliflower-like, large, malignant mass in the venous phase. It was localized in the right liver without a sharp border or capsule and recognizable macrovascular infiltration. The histology of the biopsy revealed poorly differentiated carcinoma with solid growth patterns and necrosis (Figure 3). Immunohistochemistry showed focal HepPar1 positivity in tumor cells; Arginase-1 and CK7 were negative. *TERT* mutation analysis through Sanger sequencing yielded a *TERT* mutation at 250 C > T (−146 bp). The case was signed out as poorly differentiated primary liver cancer, consistent with poorly differentiated hepatocellular carcinoma. The patient was treated with palliative chemotherapy (Atelzolizumab/Bevacizumab) but unfortunately deceased after 6 months.

Case 3 was a 53-year-old male with liver mass, peritoneal carcinosis, and unclear primary. There was a history of sclerosing cholangitis and tumor marker CA19-9 was elevated to 1198 IU/mL. CT imaging showed a new rim-enhancing mass in segment II with capsular retraction in the venous phase (Figure 2; lower panel). There were signs of direct macrovascular infiltration and peritoneal carcinomatosis. Histology revealed a poorly differentiated carcinoma with a predominantly solid, partial trabecular growth pattern and necrosis. A closer review revealed vague adenoid features (sprinkled intracytoplasmatic globules). Non-tumor tissue showed incomplete cirrhotic remodeling. Specimen staining showed single-cell tumor cells positive for CK7 > CK20; there was very weak and patchy positivity for HepPar1, and negativity for Arginase-1 (Figure 3). Next-generation sequencing analysis was performed, because imaging and clinical presentation made CCA more likely. Mutations were found in *ARID1A*, *TP53*, *PBRM1*, *LATS1* and other variants with unclear significance. The tumor mutational burden yielded 11 mutations/MB, microsattelites stable. *TERT* was reported to be wildtype (exon coverage, median > 150; fragment size, median > 70 bp). The case was signed out as poorly differentiated primary liver cancer, consistent with poorly differentiated intrahepatic cholangiocarcinoma or mixed hepatocellular cholangiocarcinoma (cHCC/CCA). The patient was lost to follow-up after 12 months.

## 4. Discussion

The practical approach to clinically uncertain, poorly differentiated liver masses has the goal of distinguishing metastasis from primary liver cancer of cholangiocytic (intrahepatic CCA) or hepatocellular (HCC) origin. If specific dynamics of contrast enhancement in imaging studies are lacking, tumor morphology must critically be reviewed for polygonal/hepatoid, pleomorph, or vaguely adenoid features, as suggested by a recent study by Kikuchi et al. [6].

The immunohistochemistry marker panel as a minimum should contain liver specific marker (HepPar1, Arginase-1) and CK7/19 [20,36]. CK20 might help to initially rule out adenocarcinoma metastasis from the gastrointestinal tract and is usually neither expressed in HCC nor CCA, although aberrant expression has been reported in both tumors [21]. HepPar1 stains normal and neoplastic hepatocytes, having a high sensitivity and specificity (>90%) [19,20,24]. Hence, there is a gradient from well-differentiated HCC (strong positivity) to poorly differentiated HCC (weak positivity or negative). Arginase-1 is the most specific and sensitive (>90%) marker for HCC, including poorly differentiated tumors and scirrhous HCC [24]. However, in our case series, Hepar1 was more helpful in diagnosis; even very faint partial staining <10% can still be considered as indicative of hepatocellular origin. CK7 (97%) and CK19 (77%) positivity generally points to a tumor origin of the pancreato-biliary tract, including intrahepatic CCA. HCC specimens can be strongly positive if they exhibit a progenitor phenotype with worse prognosis, as was first described in 2006 by Durnez et al. [22]. In this case, it is crucial to distinguish true glandular or tubular tumor formations in intrahepatic CCA or mixed HCC-CCA from *pseudo*glandular growth pattern in HCCs. The hallmarks of *pseudo*glandular growth in HCC are round, luminal structures lined with hepatocytes, but not ductal cells.

The genomic sequencing analysis of selected poorly differentiated primary liver cancer by Kikuchi et al. reported that *TERT* and *TP53* mutations as supportive for hepatocellular carcinoma. *IHD1*, *BAP1*, and *ERBB2* mutations, as well as predictive *FGFR* fusions are supportive of iCCA [6]. BRCA1-associated protein 1 (BAP1) is a tumor suppressor gene; its inactivation is observed in 25% of cholangiocarcinoma cases and (in the absence of mesothelioma) it is of potential value for differential diagnosis with very low sensitivity [25]. In the mentioned genomic analysis of poorly differentiated primary liver cancer by Kikuchi et al., 2/16 patients (each *n* = 1 with polygonal cell and indeterminate morphology) showed a *BAP1* mutation. This finding illustrates that *BAP1* mutations seem to be very rare in pd-PLC and not necessarily coherent with other findings diagnostic for CCA. In our case, we found no CCA-typical *BAP1*, *FGFR2*, *IDH1*, or *ERBB2* alterations, but we are aware of the limitations in this study, i.e., a small number of cases and a difference in sequencing methods due to restrictions in predictive testing. Nevertheless, CCA-typical *FGFR2* fusions were equally rare in Kikuchi’s pd-PLC cohort (1/16). It was recently reported that, among CCA patients, 14% are eligible for targeted *FGFR* inhibitors [37].

*TERT* mutations are rare in gastrointestinal and lung cancer but can occur in glioma and thyroid carcinoma [17,18,38]. In a Chinese cohort study of cholangiocarcinoma patients assessed by Tian et al., *TERT* alterations were observed in 21% of cases [39]. However, many of these patients had reportedly poorly or undifferentiated tumors that might as well have been poorly differentiated HCC. Further, it has been discussed that some tumors diagnosed as intrahepatic CCA in the cirrhotic liver may in fact be combined HCC/CCA due to sampling errors. The distinction between CCA and combined HCC/CCA is of relevance, because the latter is reported to reflect the mutational landscape of HCCs [40]. A technical aspect relevant to diagnosis, disease monitoring, and prognosis is the upcoming opportunities of circulating tumor (ct)−DNA in liquid biopsies [41]. Since liver cancer patients often have coagulation disorders and liver biopsies introduce many risks, considering ct DNA or microRNA-based serum biomarker might be pivotal in the future, enabling early diagnosis and prognosis [42].

In conclusion, a multidiscipline workup algorithm for poorly differentiated liver masses comprises imaging, clinical history, morphology, and immunohistochemistry to subtype poorly differentiated primary liver cancer into HCC or intrahepatic CCA. If broad-panel genomic sequencing is restricted, a closer look at polygonal morphology, faint, patchy Hepar1 staining, and single-target *TERT* sequencing can aid the diagnosis of poorly differentiated hepatocellular carcinoma.

## Figures and Tables

**Table 1:** Literature review of useful biomarkers to distinguish uncertain liver masses found in imaging.

Protein	HCC	CC	Metastasis	
HepPar-1	+	−	liver specific	[19,20]
Arginase-1	+	−	liver specific	[20]
CK19	−/+ progenitor type	+	(+) upper gastrointestinal tract/pancreatobiliary	[21,22,23]
CK7	−/+ progenitor type	+	(+) upper gastrointestinal tract/pancreatobiliary	[22,24]
CK18	+	−		[21]
CK20	−	−	(+) colorectal carcinoma	[20,21]
BAP1	preserved	lost in ~25%		[25]
Gene				
*TERT*	>70%	rare	absent in colorectal carcinoma	[6,9,26]
*CTNNB1*	30–60%	1–4%	colorectal carcinoma 80–90%	[27,28,29,30,31]
*TP53*	12–48%	24%	colorectal carcinoma 50%	[28,31,32]
*ARID1A*	4–17%	21%	colorectal carcinoma up to 67%	[7,10,31]

## Data Availability

Data are contained within the article.
